# Rural Households' Demand Status for Mitigation of *Prosopis juliflora* (*Sw.*) DC Invasion and Its Determinant Factors in Ethiopia: Empirical Evidence from Afar National Regional State

**DOI:** 10.1155/2024/5521245

**Published:** 2024-04-26

**Authors:** Kindineh Sisay, Ketema Bekele, Jema Haji, Urs Schaffner

**Affiliations:** ^1^School of Agricultural Economics and Agribusiness, Haramaya University, P.O. Box 138, Dire Dawa, Ethiopia; ^2^Head Ecosystem Management, CABI Switzerland, Rue des Grillons 1, Delémont 2800, Switzerland

## Abstract

Ethiopia is among the world's poorest nations, and its economy is growing extremely slowly; thus, the government's budget to manage environmental amenities is not always sufficient. Thus, for the provision of environmental management services such as the eradication of *Prosopis juliflora*, the participation of local households and other stakeholders is crucial. This study is therefore initiated with the objective of assessing rural households' demands for mitigating *Prosopis juliflora* invasion in the Afar Region of Ethiopia. A multistage sampling technique was employed to obtain the 313 sample rural households that were used in the analysis, and those sample households were selected randomly and independently from the Amibara and Awash Fentale districts of Afar National Regional State, Ethiopia. In doing this, a seemingly unrelated bivariate probit model was used to determine factors affecting rural households' demands for mitigating *Prosopis juliflora* invasion. Consequently, as per the inferential statistical results, there was a significant mean/percentage difference between willing and nonwilling households for the hypothesized variables, except for some variables such as farm experience; years lived in the area, distance from the market, and dependency ratio. Furthermore, the seemingly unrelated bivariate probit model result indicates that sex, family size, tenure security, livestock holding, frequency of extension contact, and years lived in the area were important factors influencing the willingness to participate in *Prosopis juliflora* management practices positively, whereas age, off-farm/nonincome, and bid value affected willingness to pay negatively and significantly. Hence, to improve the participation level of households, policymakers should target these variables.

## 1. Introduction

Invasive species are species introduced through either natural dispersion or purpose, which are the second threat to global biodiversity loss next to land use changes [1]. Globally, it is estimated that the economic cost of invasive species has been $1.288 trillion over the past 50 years [2]. Invasive alien species-induced changes cause socioeconomic deterioration such as reduction in rural households' livelihoods [3]. Furthermore, scientists have also conducted the first comprehensive study on the economic impact of a range of invasive alien species on Africa's agricultural sector, which they estimated to be USD 65.58 billion per year [4].

Of the invasive alien species, *Prosopis* is the one that is indigenous to South America, the Caribbean, and Central America [5]. Among the different highly invasive *Prosopis* species, *Prosopis juliflora* (*Sw.*) *DC* (hereafter called *P. juliflora*), which is a member of the Fabaceae family, is the only one that was found in Ethiopia. However, the exact date and source of its introduction to Ethiopia have not been clearly documented. However, it was believed to have been introduced in India in the late 1970s by the Ministry of Agriculture intentionally for conservation [6–8]. Since then, the tree has rapidly invaded vast areas of pastoral and agropastoral lands in the Afar Region and some other parts of the country [9]. In 2019, the areas of land that have been invaded by *P. juliflora* reached 1.2 million [9]. The presence of *P. juliflora* in rangelands leads to large-scale economic losses in the form of reduced levels of animal productivity, increased herd mobility rates, and more difficult stock handling [10–15]. Moreover, in those studies, it was also indicated that the aggregate loss due to *P. juliflora* far outweighed its ecological benefits and the local people were bitter about its introduction.

Regarding the economic effects that *P. juliflora* imposed in the Afar Region of Ethiopia, literature shows that it absorbs roughly 3.1–3.3 billion meter cubes of water annually that could irrigate 460,000 hectares of cotton and 330,000 hectares of sugarcane, yielding an estimated net benefit of about US$320 million and US$470 million from cotton and sugarcane, respectively, per growing season [16]. Yet, the examination of policy and stakeholders' analysis for managing invasive plants in Ethiopia revealed that the institutional mandate is hazy and fragmented, and the so far implemented interventions have not been proactive or effective [17]. To sum up, research on *P. juliflora* management and the impact of *P. juliflora* has been extensive in the study area, e.g., [10–15, 18–22]; thus, different management practices have been developed, yet their successes are very limited. This is because the effectiveness and sustainability of any management intervention in general require the full participation and willingness of local communities for the fact that they are the immediate victims of the negative effects caused by the species. Thus, this study is carried out to distinguish factors that hinder households' demand to mitigate the invasion of *P. juliflora*, thereby filling the existing literature gap.

Ethiopia is among the world's poorest nations, and its economy is expanding extremely slowly; thus, the government's budget to manage environmental amenities is not always sufficient. Thus, for the provision of environmental management services such as the eradication of *P. juliflora*, the participation of local households and other stakeholders is crucial. As the invasion of *P. juliflora* has numerous detrimental effects on the livelihood of rural families, it is becoming obvious that these households, together with other interested parties, should bear some of the financial burden of mitigating these issues through their participation in *P. juliflora* management practices. By doing this, it will become clear what steps need to be taken to implement sustainable and participatory management practices by looking at the factors that influence rural households' willingness to pay or donate in kind to the management of *P. juliflora*. In addition, the study will be significant from the perspectives of forthcoming researchers, policymakers, and the environment. First, it is anticipated that the study's findings will significantly influence the mobilization of families that are willing to engage in *P. juliflora* management, ultimately leading to the sustainable management of this IAS. Additionally, to create an efficient plan and handle the issues or limitations with *P. juliflora* management services, policymakers at the national or regional levels are anticipated to take some lessons from this. Above all, since this study is the first of its type in the field, it will serve as a benchmark for forthcoming researchers who are interested in the same or comparable topics in other parts of Ethiopia or elsewhere in the world where *P. juliflora* was introduced.

The rest of this study is organized into four main sections. The next section presents the literature review, where the theoretical review, empirical review, and conceptual framework emanating from the review are presented concisely. This was followed by the materials and methods section, in which a brief description of the study area, types/sources/methods of data collection, the sampling procedure and its determination, and the estimation methods are deliberated. Thus, the detailed interpretations, discussions, and justifications are presented in the subsequent section. Finally, the last section concludes the study by presenting the main conclusions and recommendations emanating from the findings.

## 2. Literature Review

In this section, a theoretical review including the definition, spread, uses, and adverse effects of *P. juliflora* is presented. This was followed by a general empirical review of natural resource valuation studies that have been conducted in Ethiopia and elsewhere using the contingent valuation method. Then, studies on *P. juliflora* management in the study area were reviewed. Finally, the conceptual framework of the study is presented in a summary figure.

### 2.1. Theoretical Review

In general, invasive alien species (IAS) can be defined as species that can establish themselves outside their natural range and, once established, rapidly extend their range in the new region, causing significant harm to biological diversity, ecosystem functioning, and human health in the invaded region [23]. Invasion of woody weeds into range and agricultural land is becoming a problem in major regions worldwide [24–27]. Invasions can be classified depending on whether they occur in the native range or where they have been introduced [28–30]. *P. juliflora* is defined as any of several small spiny trees or shrubs having small flowers in axillary cylindrical spikes followed by large pods rich in sugar. The name *juliflora* comes from julus, meaning “whip-like,” referring to the long inflorescences, and flora being the flower [5]. It is among the most common tree species to be found in the dry tropics.

The first records of *Prosopis* introduction are those to West Africa and Pacific Islands in or before the 1820s, to India and Pakistan in the 1870s, and to Australia and South Africa before 1900. However, there have been many other unrecorded introductions before and since, as evident by the fact that *Prosopis* is now found in the dry regions of most African and Asian countries [5]. Of all the *Prosopis* introduced, only *P. juliflora* and *P. pallida* are now naturalized wherever they have been introduced and are by far the most common *Prosopis* species in tropical desert regions. As trials show, *P. pallida* is generally faster-growing, more erect in habit, and less thorny than *P. juliflora*. The exact date and source of *Prosopis* introduction to Ethiopia had not been clearly documented; however, it was believed to have been introduced in India in the 1970s by the Ministry of Agriculture for conservation purposes [6].

In recent decades, these “exotic” *Prosopis* have attracted much attention. They are extensively planted as fast-growing and drought-tolerant fuel and fodder trees, but in many countries, they spread out of control as invasive weeds. However, as they grow wild and in abundance on common lands, they are especially important to poor farmers and the landless [23]. *P. juliflora* produces abundant quantities of often sweet fruit pods, readily consumed by all livestock and wild animals. Hence, it is thought to be a valuable fodder because it can be either browsed or collected, fed completely, or processed as a feed for all livestock from chickens to camels [23].

*P. juliflora* can survive on inhospitable sites where little else can grow, tolerating some of the hottest temperatures ever recorded and, on poor, even very saline or alkaline soils. As they are nitrogen fixers, *P. juliflora* have also been noted to improve the fertility and physical characteristics of the soils in which they grow. They have deep roots, allowing trees to reach water tables and bear fruit even in the driest years, providing an invaluable buffer during droughts. However, the aggregate loss due to *Prosopis* far outweighs these ecological benefits, and the local community members are bitter about the introduction of *Prosopis* [10, 11]. Due to this, its invasiveness becomes a researchable and attention-seeking issue because of its adverse effects, which will be discussed later.

There are reports from almost every country where they are introduced of exotic *Prosopis* invading agricultural and pasture land, nature reserves, waterways, roadsides, and wasteland [5]. The stout thorns of some species and the tendency to become bushy in form when cut or browsed can lead to the formation of impenetrable thickets. According to Wakie et al. [21], over the past four decades, the number of livestock owned per household in the seven studied villages of Afar has declined by more than 50%. Among the main reasons reported for the decline of their livestock assets, the invasion of *P. juliflora* is the prime one.

Both men and animals dislike the thorns, and this makes *P. juliflora* a particularly undesirable weed [23]. The thorns of the species are pairs, commonly 1–5 cm long and with a thick base; however, some species have thorns over 20 cm long, while others are thornless [6]. A broken branch on the ground will always have thorns pointing upwards, which are able to pierce car tires and are a danger to the feet of all animals. *P. juliflora* pollen has also been reported to cause allergic reactions. In addition to this, *P. juliflora* also negatively affects the native flora by invading grasslands, shrublands, and woodlands. The most affected useful native grass and herb species in Ethiopia include *Chrysopogon* spp. (durfu), *Eragrostis* spp. (denikto), *Setaria* spp. (delaita), *Cenchrus* spp. (serdoita), *Hyparrhenia* spp. (isisu), *Cynodon* spp. (rareita), and *Andropogon* spp. (melif), while useful native tree species mostly affected include *Combretum aculeatum* (kilito), *Acacia tortilis* (ehebto), and *Acacia nilotica* (keselto) [21].

### 2.2. Empirical Review

#### 2.2.1. General Review

This investigation attempted to review different natural resource valuation studies that have been conducted using the contingent valuation method (CVM) both in foreign countries [31–34] and in Ethiopia [35–40] using a systematic review. Then, studies that have been conducted on *P. juliflora* management in the study area (Afar Region) were also reviewed. Consequently, almost all of the above-reviewed willingness-to-pay (WTP) studies were conducted using cash contribution as a payment vehicle. Due to the payment vehicles chosen (cash contribution), the variable household income was found to affect WTP significantly and positively in almost all of the reviewed studies. Furthermore, the variable education level of the household head was also found to affect WTP significantly and positively in most of the reviewed studies. Despite this, in almost all of the above-reviewed studies, income, education level, and bid price were included as explanatory variables, whereas age, sex, and household size were also included as explanatory variables in most of the reviewed studies. From the reviewed studies, it was found that every study that used household size as an explanatory variable also found a substantial negative relationship with WTP. The associated reason for this was the payment vehicle chosen (cash contribution). This is because the variable household size was observed only from a consumption perspective when the payment vehicle was in cash. This reveals that if the payment vehicle chosen is not in labor contribution, then the household could not consider their household size from a labor contribution perspective.

#### 2.2.2. Empirical Studies on *P. juliflora* Management in Afar

The study by Tilahun et al. [41] investigated the practices to mitigate *P. juliflora* invasion in three districts of Afar. According to the findings of this study, approximately 84% of respondents favor a total eradication of *P. juliflora*. The median willingness to make a contribution to this intervention was 9.97 and 13.42 USD/household/year. The study further revealed that off-farm income and *P. juliflora* invasion levels on pasturelands are among the factors influencing willingness to contribute to *P. juliflora* invasion mitigation. The study emphasized the need to provide incentives to local residents as well as establish a solid institutional architecture that includes local culture and institutions for mobilizing people on a voluntary basis to combat *P. juliflora* invasion.

Another study was also conducted by Ilukor et al. [20] on “to eradicate or not to eradicate recommendations on *P. juliflora* management in Afar, Ethiopia, from an interdisciplinary perspective.” This report presents the outcomes of a multidisciplinary study on the proliferation of *P. juliflora* as an invasive species in the Afar Region, along with recommendations for its management and control. Socioeconomic data were collected from pastoral households in Amibara, Gewane, and Awash Fentale. The research revealed that wetlands, particularly the floodplains of the Awash Basin, are more susceptible to *P. juliflora* invasion than dry lands. It is proposed that clearing invaded areas and consistently using them for agriculture could help limit the spread of invasive species. The study has identified key aspects of *P. juliflora* invasion in the Afar Region and suggested that sustainable management and control methods are the most effective approaches.

Another study was conducted by Wakie et al. [21] on household-level preferences for the mitigation of *P. juliflora* invasion in the Afar Region of Ethiopia. The study's goal was to analyze the economic feasibility of specific *P. juliflora* eradication and usage options currently applied in one of the most suffering regions of Ethiopia, which is Afar. Conversion of *P. juliflora*-infested fields into irrigated agriculture, charcoal production, and seed flour production are among the options used. By interviewing 19 business owners, the researchers calculated the costs and revenues of the selected *P. juliflora* eradication and utilization options. As the result suggested, converting to irrigated cotton is economically viable, with a net present value of 5234 US$/ha over ten years and a 10% annual interest rate. *P. juliflora* dispersion on farmlands is considerably reduced by conversion. It is also shown that maintaining *P. juliflora*-infested fields for charcoal production with a four-year harvest cycle is viable, with an NPV of $805 US$/ha. Conversion and charcoal production may be carried out with little investment. Finally, the study indicated that given the correct environmental conditions, control by usage is a feasible *P. juliflora* management technique.

A study by Nigussie et al. [19] on the analysis of the invasion rate, impacts, and control measures of *P. juliflora* was also undertaken in the study area. This study investigated the dynamics and consequences of *P. juliflora* invasion, as well as the efficacy of current management strategies. According to this study, the high invasion rate can be attributed to the seed's high germination rate, seed dispersal mechanisms, and wide-ranging ecological adaptation. Because of the restricted spatial size, expense, and/or incorrect design and implementation, the management procedures that have been established are unable to produce the desired results. As a result, within the scope of the existing villagization initiative, the design of a strategy for management measures that involve community engagement and minimize the number of vector animals was revealed as a critical point. Furthermore, the study suggested conducting a risk assessment before introducing an alien species into a particular area.

### 2.3. Conceptual Framework

**Figure 1:**
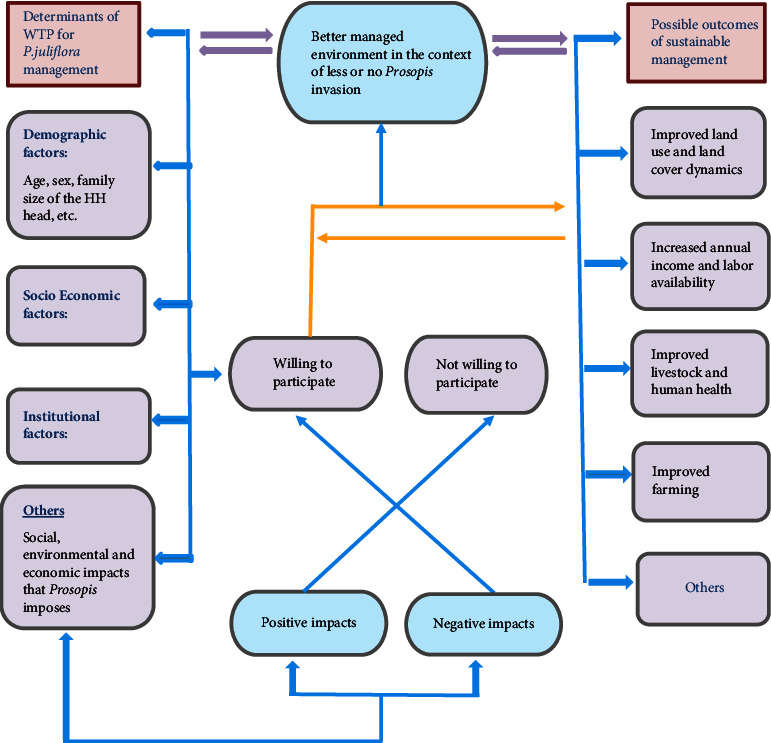
Conceptual framework of the study. Source: own construction based on the literature reviewed (2021).

For the willingness of households to participate in *P. juliflora* management practices, there are different possible determinants or factors that can affect their willingness, which include demographic factors (age of the HH head, sex of the HH head, family size, etc.), socioeconomic factors (total farm income, off-farm income, livestock holding, size of own land, etc.), and institutional factors (extension contact, tenure security, etc.). All these factors can affect rural households' willingness to participate in *P. juliflora* management either directly or indirectly. In addition to all these determinants, there are also other factors that can influence their willingness to participate from the amenity side of *P. juliflora*, which include social, environmental, and economic impacts that *Prosopis* can impose on the household. If the positive impact that *P. juliflora* can impose on the household is greater than the negative one, then the household will not be willing or less willing to participate in the management practices implemented by the Woody Weed Project, and those households whose negative impact from *P. juliflora* is outweighing the positive one will be more willing to participate in these management practices. When the households are willing to participate in the management practices, then there will be a better-managed environment in terms of reduced invasion. This better-managed environment can lead to improved farming, increased annual income and labor availability, improved land use and land cover dynamics, improved livestock and human health, etc. This situation and other additional concepts are represented in the pictogram form of the conceptual framework (Figure 1).

## 3. Materials and Methods

### 3.1. Brief Description of the Study Area

**Figure 2:**
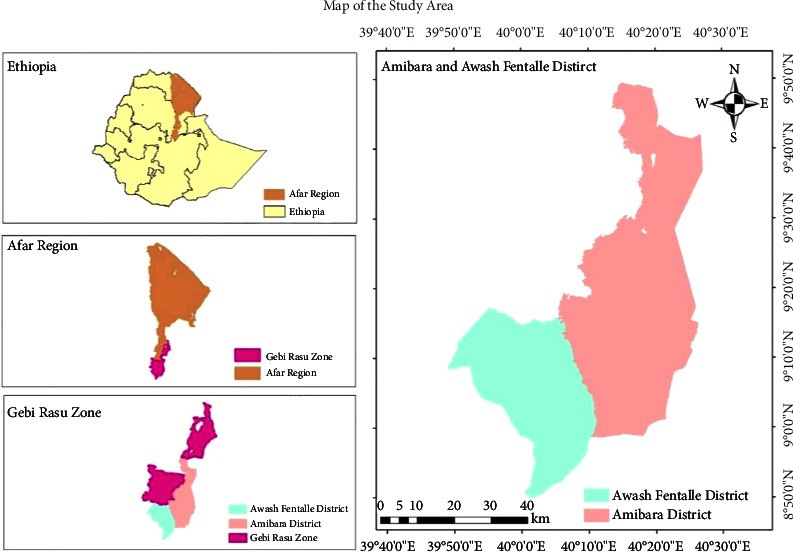
Map of the study area. Source: own ArcGIS mapping (2021).

Amibara and Awash Fentale are the districts in the Afar Region of Ethiopia that are part of administrative zone 3 (Figure 2). Amibara is surrounded by administrative zone 5, Gewane, the Somali Region, the Awash Fentale in the south, and the Awash River in the west, which divides it from Dulecha, the Awash River in the northwest, and the Oromia Region in the southeast. The Oromia Region borders Awash Fentale on the south, the Amhara Region on the west, Dulecha on the north, and Amibara on the east. Based on the 2007 census population projection conducted by the central statistical agency of Ethiopia [42], Amibara and Awash Fentale had a total population of 76,649 and 29,780, respectively.

### 3.2. Types, Sources, and Methods of Data Collection

To come up with the results of the study, both qualitative and quantitative types of data were used. For obtaining those data, both primary and secondary sources of data were castoff. The primary data that were utilized in the descriptive and empirical analysis of this study were collected using key informant interviews, focus group discussions, and a structured questionnaire from sample households. The primary data and/or contingent valuation survey that were collected using structured questionnaires were administered by more experienced and trained enumerators from January 15 to February 5, 2021. Secondary data were collected from different sources such as the district agricultural office, population census records, journal articles, websites, books, and magazines to supplement the study.

The contingent valuation technique (CVM) is one of the most extensively used stated preference methods. It is a “generic approach” [43] that replicate genuine market circumstances to elicit individuals' preferences for a specific environmental item. This method employs a survey to directly ask participants how much they are WTP for a welfare gain (due to increased utility) or how much they are willing to accept (WTA) as compensation for a welfare loss (due to decreased utility) that occurs as a result of a change in the specified environmental item [44], or how much better/worse off individuals are or will be as a result of a change in environmental quality. According to [45], the CVM constitutes the only alternative to attaining economic value estimates when there is a presence of distortions in environmental goods and services, and there are no effective or substitute markets for them. It is also pointed out that one of the advantages of this type of methodology is that it results in precise estimates of values that cannot be obtained by other ways [46].

Under CVM, double-bounded dichotomous (take it or leave it with follow-up) elicitation methods were employed to elicit respondents' WTP. It is used in such a way that a respondent is offered a follow-up question based on his or her response to the first inquiry. The first question is followed by another question that specifies a smaller amount if the first question was answered no and a bigger amount if the first question was answered yes. This indicates that under the double-bounded model, the interval is bound by two bids. The first bids are changed across persons to obtain additional information and for genuine WTP distribution [47, 48]. Some people may be unwilling to engage in the planned intervention because their valuation is between zero and a certain amount less than the recommended bid amount [49]. References [50, 51] conclude that adding a follow-up bid to a conventional dichotomous choice contingent valuation survey can significantly improve the efficiency of the discrete-choice questionnaire.

In at least three aspects, the double-bounded dichotomous choice format is superior to the single-bounded format for correcting strategic bias and improving statistical efficiency. First, it is comparable to the existing market system in Ethiopia, in which vendors specify an initial price and purchasers are given the opportunity to bargain [52]. Second, in the double-bounded dichotomous choice format, the yes-yes, no-no answer sharpens the real WTP and establishes unambiguous limitations on unobservable WTP; hence, there will be efficiency benefits [49]. Finally, the double-bounded dichotomous choice format is more efficient than the single-bounded dichotomous choice format because it elicits more information on each respondent's WTP and allows for the estimation of a parametric mean [47, 49].

### 3.3. Sampling Procedure and Sample Size Determination

Multistage sampling methods were employed to gather available information from the rural households of the study districts (Figure 2). In the first stage, the Afar Region was selected purposefully as it is the most heavily invaded region in the country. Furthermore, the Gabi Rasu zone (zone 3) was also purposefully selected as it is the most *P. juliflora*-invaded area of the region where agricultural productivity is declining. Based on a panel of experts' discussions and observations during the pilot test, the districts were categorized into two levels of invasion (strata). Accordingly, Amibara, Gewane, and Gala'alu are the highly invaded districts, while Awash, Fentale, Dulecha, and Argoba are the less invaded districts. Then, Amibara and Awash Fentale districts were randomly selected from each invasion category (Figure 2). Secondly, 4 *kebeles* from each study district that had different invasion levels were selected from the Amibara and Awash Fentale districts with the consent of community representatives and district experts on this issue. Lastly, 162 and 161 household heads (a total of 323) were randomly and independently selected from the Amibara and Awash Fentale districts, respectively, based on each selected *kebele* of the study districts using the probability proportionate to size technique. In doing this, the minimum sample size required for the study was determined using the Yamane [53] sample size calculation method, which is the best method for a homogeneous population.

The formula is given by

(1) $$\begin{array}{c} n=\frac{N}{1+N\left(e^{2}\right)}, \end{array}$$

where *n* represents the required sample size; *N* represents the population size or total number of households in the district; and *e* represents the level of precision, which is assumed to be 7.8% in this study.

### 3.4. Methods of Data Analysis

#### 3.4.1. Descriptive and Inferential Statistics

Descriptive statistics such as mean, percentage, and standard deviation were used to show different demographic, socioeconomic, and institutional characteristics. In addition, the *t*-test and chi-square tests were employed to show the effect of the hypothesized explanatory variables on the WTP. While doing this, the *t*-test was used to check the existence of the mean difference between continuous/discrete independent variables across willing and nonwilling households. However, the chi-square test was employed to illustrate the association between dummy/categorical explanatory variables and the dependent variables.

#### 3.4.2. Identifying Factors Affecting Willingness to Pay

The goal is to estimate the correlation between personal traits and the likelihood of households' WTP given an initial bid value chosen at random. Farmers have the option to accept or reject the predefined offer for a given specified amount of labor days that must be deducted from a specific household's labor endowment for *P. juliflora* management for the dichotomous choice question of the CVM survey. According to [54], a simple utility framework was used to model how rural households make decisions. Let us say a rural household's utility or satisfaction is determined as follows:

(2) $$\begin{array}{c} U_{i}=U_{i}\left(L,Z,q\right), \end{array}$$

where *U*_*i*_ is the utility of the household *i*, *L* is the total labor endowment of the household in a year, *Z* is the demographic, socioeconomic, and institutional characteristics of the household, and *q* is *P. juliflora* invasion level as perceived by the farmer. Furthermore, let us assume that there are two states of the world corresponding to different levels of *P. juliflora* invasion: *q*^*∗*^ as the invasion level after the management practices are undertaken and *q* as the invasion level before the *P. juliflora* management practices are undertaken or if the practice is not pursued.

As the total labor endowment availability of a particular household is a principal or most limiting asset of the household, it is assumed that the individual will be willing to contribute to the suggested reduction in its total labor endowment to maximize his or her utility under the following conditions or reject it otherwise:

(3) $$\begin{array}{c} U_{i}^{1}\left(L-BID,Z,q^{*}\right)+e_{1}\geq U_{i}^{0}\left(L,Z,q\right)+e_{0}, \end{array}$$

where *U*_*i*_, *L*, and *q*^*∗*^ are as defined above, BID is the initial labor day contribution per year for *P. juliflora* management practices, and *e*_1_ and *e*_0_ are the error terms, which are with zero means and independently distributed. Therefore, the probability that a household will decide to contribute to *P. juliflora* management is the likelihood that the conditional indirect utility function for the planned program is greater than the conditional indirect utility function for the status quo. It is worth stating that the utility functions are generally unobservable. The utility function of the *i*^th^ family that is expected to be a function of noticeable family features, resource endowment, and ecological excellence *X*_*ti*_ and a disturbance term *e*_*ti*_ can be specified as follows:

(4) $$\begin{array}{c} U_{i}^{t}=f\left(X_{ti}\right)+e_{ti},t=0,1\;i=1,2,\ldots \;n. \end{array}$$

The focus in this model is on the factors that determine the probability of accepting the initial bid. The *i*^th^ farm household will be willing to accept the initial bid when  *U*_*i*_^1^ ≥ *U*_*i*_^0^. Therefore, the choice problem can be modeled as a binary response variable *Y*, where

(5) $$\begin{array}{c} Y_{i}=\left\{\begin{array}{cc} 1, & if\;U_{i}^{1}\left(R_{e}-BID,Z,q^{*}\right)+e_{1}\geq U_{i}^{0}\left(R_{e},Z,q\right)+e_{0},\\ 0, & \text{otherwise}\ldots \ldots \ldots \ldots \ldots \ldots \ldots \ldots \ldots \ldots \ldots \ldots \ldots \ldots \ldots \ldots \ldots , \end{array}\right. \end{array}$$

The probability that a given household is willing to contribute to *P. juliflora* management is given by

(6) $$\begin{array}{c} \text{Prob}\left({\;Y}_{i}=1\right)=\text{Prob }\left(U_{i}^{1}>U_{i}^{0}\right). \end{array}$$

Through substitution, we will have

(7) $$\begin{array}{c} \text{Prob }\left(Y=1\right)=\text{Prob}\left(\alpha_{1}X_{i}+e_{1i}>\alpha_{0}X_{1}+e_{0i}\right). \end{array}$$

By rearranging equation (7), we obtain

(8) $$\begin{array}{c} \text{Prob}\left(Y=1\right)=\text{Prob}\left[\left(e_{1i}{-e}_{0i}\right)>X_{i}\left(\alpha_{0}-\alpha_{1}\right)\right]. \end{array}$$

If we assume *U*_*i*_=*e*_1*i*_−*e*_0*i*_ and *β*=*α*_0_ − *α*_1_, we will have

(9) $$\begin{array}{c} \text{Prob}\left(Y=1\right)=\text{Prob}\left(U_{i}{>X}_{i}\beta \right)=F\left(X_{i}\beta \right), \end{array}$$

where *F* represents the cumulative distribution function. This provides an underlying structural model for estimating the probability and it can be estimated using either a probit model or logit model, depending on the assumption of the distribution of the error term (*e*) and computational convenience. Consequently, following the work of [32–34, 38–40] that followed the contingent valuation method, this study employed the seemingly unrelated bivariate probit (SUBP) model by introducing independent variables that were hypothesized as an influencing factor in addition to the bid variable. Assuming a normal distribution of the error terms, the seemingly unrelated bivariate probit model can be specified as

(10) $$\begin{array}{c} y_{1}^{*}=a_{1}+\beta_{1}t_{1}+\overset{n}{\underset{i=1}{\sum }}\beta_{i}x_{i}+e_{1},\\ y_{2}^{*}=a_{2}+\beta_{2}t_{2}+\overset{n}{\underset{i=1}{\sum }}\beta_{i}x_{i}+e_{2}, \end{array}$$

where *y*_1_^*∗*^ and *y*_2_^*∗*^ are the *i*^th^ rural households' WTP for *P. juliflora* management when she/he responds to the initial and subsequent WTP questions, respectively; *t*_1_ and *t*_2_ are the initial bid and the second bid, respectively; *a* and *β* are unknown parameters to be estimated; *e*_1_ and *e*_2_ are unobservable random components distributed *N* (*0*, *σ*); and *x*_*i*_ refers to the independent variables. Generally, the independent variables (*x*_*i*_) are the same in the two equations above other than the bid variables (bid_1_ and bid_2_).

### 3.5. Definition of Variables and Hypothesis

The dependent variables are binary choice variables measuring the willingness of rural households to participate in *P. juliflora* management practices using labor contribution. The value of the dependent variable is 1 for the “yes” to the initial bid and zero otherwise and the same way for the second bid. With market imperfection, the probability or the level of farm households' WTP for nonmarket goods management and investment decisions depends on various factors including household characteristics. Depending on the different theoretical evidence and findings of past studies, the following variables (Table 1) were hypothesized for this study to determine households' WTP for *P. juliflora* management.

## 4. Results and Discussion

### 4.1. Descriptive Results of Continuous Independent Variables

Households in the two categories were found to be statistically different in terms of their age, education, farm income, nonfarm income, extension contact, size of own land, family size, dependency ratio, TLU, and initial bid. For instance, the mean age of the willing and nonwilling respondents was 44.41 and 58.20 years, respectively (Table 2). A comparison between willing and nonwilling respondents based on the mean age indicates that those nonwilling household heads are older than those willing respondents. The respective independent *t*-test results show that the mean age difference between willing and nonwilling respondents is statistically significant at the 1% probability level. Moreover, the mean number of years a household head spent at school was also computed, and surprisingly, it was found to be very low, with a mean educational level of grade 1 (0.44), which is too less compared with the national average of 2.9 years [55]. A comparison between willing and nonwilling respondents based on the mean number of years spent in school indicates that unpredictably those nonwilling household heads spent more years (2.15) than those willing respondents (0.32) (Table 2). The entire sampled households had an average of 4.23 family members in the adult equivalent, which is almost equal to the national average of 4.6 members [56]. The mean household size of willing and nonwilling households was 4.34 and 2.72, respectively, and the difference was statistically significant at the 1% probability level (Table 2).

### 4.2. Descriptive Results of Dummy Independent Variables

Following the work of [40, 57–65], the chi-square (*χ*^2^) test was used to check the (mean difference) existence of variation in terms of various dummy independent variables among the two groups of WTP status. Accordingly, there exists a statistically significant mean difference (at 1%) in sex, tenure security, and past awareness between the two groups. For instance, it was shown that households who were sure about their land security (secured that their land would be with them) were found to be more willing to participate in the intervention than their counterparts. Of the total secured households (85), all of them were willing to contribute labor for *P. juliflora* management (Table 3). As the chi-square (*χ*^2^) result depicts, there was a significant mean difference in the willingness to participate status between secured and nonsecured households at the 1% probability level.

### 4.3. Determinants to Participate in the Mitigation of *P. juliflora* Invasion

After distinguishing willing and nonwilling households for mitigating *P. juliflora* invasion and determining the presence of differences among households in their demand, finding out the factors causing this disparity among rural households was the next most important step of this study. To see the demand/willingness responses of the sample households, the dependent variable (WTP) was regressed on factors that were expected to affect the demand for mitigation of the species using the SUBP estimation procedure as presented as follows (Table 4).

As the model result reveals, the age of the household head has a negative influence on the WTP of the households to contribute labor for *P. juliflora* management, which is consistent with the prior expectation. Thus, keeping the effect of other factors constant at their mean value, a one-year increase in the age of the household head decreases the probability that the household could contribute by about 0.41% and it was found to be significant at the 1% probability level for the second response. Such a negative and significant relationship between the age of the household head and the WTP amount might be allied with the labor constraints (inability to participate in such labor-intensive activities) of the old-aged households compared with the old- and middle-aged households. However, as older household heads have shorter planning horizons, they expect that they will benefit less from the investment relative to young household heads, given that the benefits are generally longer term in nature (based on the proposed study, a giant benefit is expected after five years). Hence, this labor constraint and having a shorter planning horizon negatively affect WTP. The result was consistent with the hypothesis and the findings of [40, 57–60].

As a prior expectation, the sex of the household head was positively related to willingness to participate in *P. juliflora* management and it happened to be significant at the 10% probability level for the first response. The marginal effect of the variable indicates that keeping other factors constant, being male increases the probability of participating in *P. juliflora* management by about 0.71%. This is mainly because female-headed households have some cultural constraints compared with male-headed households. In addition to this, being in a female-headed household means most probably not having a husband (being widowed or divorced). Therefore, those female household heads are going to cover both internal and external duties so that they are not going to get free time and labor to contribute to the intervention. Hence, those female-headed households are going to be nonwilling to participate in *P. juliflora* management. References [57, 61–64] reported the same result in the magnitude of sex for WTP studies in environmental or natural resource management.

Family size was found to be significant for the second response at the 1% probability level and to be related to the respondents' willingness to participate positively. Keeping other factors constant, when the family size of the household increases by one unit in adult equivalent, the number of labor days a household is willing to contribute for *P. juliflora* management will also increase by 4.21%. This result is in line (in both magnitude and argument) with the findings of [57, 66]. The study result contradicts the findings of [31, 37, 60, 67]. For these contradictory studies, their common point of argument is that an increase in family size means more expenditure on other things and decreases the per capita income of the members, hence decreasing the participation in environmental or natural resource management. Moreover, having larger family members is related to more consumption without or with less labor contribution by each household member for farming or other activities; thus, the household head becomes nonwilling. Consequently, the main reasons for this contradiction are the difference in the level of education of children and the difference in the payment vehicles chosen. As the survey result indicates, in the study area only 42% of the households were educating at least some of their children, whereas in other regions of Ethiopia, almost all households educated at least some of their children. Hence, in the study area, it is better to look at family size from a labor contribution perspective rather than from a consumption perspective. Therefore, a larger family size means more labor availability in the household, which will have a positive relationship with WTP.

Off/nonfarm income was found to affect rural households' WTP negatively and significantly. Holding other factors constant, as the annual off/nonfarm income of the household increases by one ETB, the amount that the household could contribute will decrease by 2.64*e*^−04^% at the 1% probability level for both responses. In line with this study, [40, 68–70] confirmed that an increase in nonfarm income could decrease household dependence on agricultural activities and values derived from them. According to these studies, such a decrease in reliance on agriculture negatively affects households' WTP because farming is not their main income-generating activity. For this study, one of the possible reasons is similar to those inferred from previous studies. In addition to this, those nonfarm income-generating households/members were generating their nonfarm income from charcoal production and firewood selling and the plant species used for such purposes were *P. juliflora*. Due to this, they are going to be nonwilling to participate in *P. juliflora* management practices. Despite this, the study contradicts the findings of [41], whose main point of argument is that having more nonfarm income could solve the financial constraint and encourage them to pay more money. This suggests that differences in the payment vehicle chosen and the relationship of nonfarm activity/activities with the proposed intervention are the main reasons for such contradiction with the study at hand.

Holding other factors constant, having secured land, or having a land certificate increases the probability of households' WTP by 7.05% compared with those who do not have secured land at the 1% probability level for response. Hence, those households that think that the land they own is secured tend to contribute more to *P. juliflora* management than their counterparts. This is because having secured land creates trust between the household and the government for future possession of their land. As a result, tenure security can be positively associated with a higher level of contribution to the proposed intervention (*P. juliflora* management).

The FGD result confirms that in most parts of the Afar Region, the land was owned by the *Gosa* (which is a clan of families who live in the same area with groups and this Gosa has its own representatives who have the power to undertake any decision by representing the entire members) and only the representative of the *Gosa* was responsible for sharing or giving land in need for their members or relatives. If the available land that was owned by the *Gosa* became slackened or denuded, those representatives would try to redistribute the land they had previously overdistributed. Therefore, due to its higher tension and frustration, even if households or household members obtain land from their *Gosa*, they are not going to be involved in cultivating and managing the land they have been given, and they do not show possession rights; hence, the land remains unoccupied. The associated reason is that households worry about the redistribution of the land they have been given by their *Gosa* representatives in particular and by the government in general. Furthermore, while giving or distributing the land to the household head or members, the *Gosa* leaders are not given the right to them in order to use it in accordance with the property right guides (transferable, attainable, enforceable, etc.). Due to all these reasons, almost all the available land in the study area remained unoccupied except for grazing and hence remained invaded by *P. juliflora*, signifying that those households who think that the land they own is not secured (those households who do not have a land certificate) have a lower probability of WTP than the secured households. This study is also consistent with the hypothesis and previous studies of [57, 71–73].

Livestock holding measured in the tropical livestock units was found to have a significant and positive influence on households' WTP. Thus, a one-unit increase in livestock holding in TLU increases the amount that the household could contribute by 0.14% and it happened to be significant at the 1% probability level for both responses. In the study area, livestock rearing is a proxy for households' wealth and serves as a main source of income (77.6%). Moreover, households in the study area are almost pastoralists (64.5%), and even for those agropastoralists, their main source of income was livestock production; hence, the communal land they have and other free areas of the region serve as the main source of feed for their livestock rather than using the land for other purposes. As a result, more livestock holders in TLU might be more willing to participate in *P. juliflora* management to maintain their livestock health, reduce the risk of predators, and minimize the risk of feed loss (to minimize grazing land deterioration) for such a large number of livestock they own. This argument was also supported by the results obtained from KII (from Adilao Aramis, a model farmer in *Halaydege Kebele*, Amibara District: “We are losing our large number of livestock by the consequence of *P. juliflora*. Over consumption on the seed of the plant can leads to bloating, prick of its thorn can also expose to infection and as well it exposes to predator because, when we lost our livestock, the plant does not allow us to freely move and search the livestock we loosed. Even in this week, I have loosed five sheep's and I did not found them still now. Therefore, to improve the livelihood of my household through livestock production, I am highly interested to participate in *P. juliflora* management.”), which raises different consequences of the plant on the livestock resource and thereby its implications for a large number of livestock owners to become more willing to participate in *P. juliflora* management. Therefore, households with more livestock holdings are positively and significantly associated with a higher level of contribution to the management of the species. This study is consistent with previous studies by [40, 41, 57, 58, 74].

Extension visits, which are the primary source of information related to natural resources or environmental management, were found to have a positive and significant effect on the WTP in *P. juliflora* management. Therefore, an increase in extension contact by one more visit increased the households' WTP by 0.40% and it happened to be significant at the 5% and 10% probability levels for the first and second responses, respectively, ceteris paribus. The possible reason is that having more extension contact is always associated with an enhancement in households' awareness regarding the invasion level of *P. juliflora* and its consequences. Hence, frequent extension visits about natural resources or environmental management in general and *P. juliflora* management in particular could inspire households to participate in *P. juliflora* management. In line with this finding, [58, 69, 75–77] also emphasized that extension contact enhances households' awareness of managing environmental or natural resources, and this can positively affect their WTP.

Consistent with prior expectations, years lived, measured in the number of years, were found to have a significant and positive influence on households' willingness to contribute to *P. juliflora* management. Thus, holding other factors constant, a one-year increase in the number of years living in a particular area increases the amount that the household could contribute by 0.21% at the 1% probability level for the second equation. One of the possible reasons for this is that as the household lives for a long time in a particular area, they feel more responsible and accountable for the safety of the area, and they are living longer. Therefore, to surmount their responsibility and accountability, households will be more willing to participate in *P. juliflora* management.

As a prior expectation, the bid value (bid_2_) was found to have a negative and statistically significant influence on the WTP. This negative relationship signifies that an increase in the offered bid amount could lower the probability of households' willingness to participate in *P. juliflora* management. Keeping the effect of other factors constant, when the bid value/bid_2_ increases by one labor day, the probability of households' WTP in *P. juliflora* management decreases by 0.24% at the 5% probability level for the second response. This result is consistent with the theory of demand, which articulates that demand increases as the price decreases and vice versa. Therefore, households who offered a higher bid amount were more likely to be nonwilling to participate in *P. juliflora* management than those who offered a lower bid amount. The result is inconformity with the results of the studies conducted by [36, 37, 40, 60, 78, 79].

## 5. Conclusions and Recommendations

### 5.1. Conclusions

Currently, IAS is invading large areas of the Afar Region and it gives rise to social, economic, and environmental impacts on the rural households living in the region. Therefore, to improve the livelihood and welfare of those rural households, *P. juliflora* management is found to be a critical decision. Hence, the current study, which assessed factors affecting rural households' demand for mitigating the invasion of *P. juliflora* was considered a bouncing step. Data collected from 313 sample respondents were analyzed using descriptive statistics, inferential statistics, content analysis, and an econometric model to meet the desired objective of the study. The result of descriptive statistics showed that 293 (93.61%) households were found to have a demand/willingness to mitigate *P. juliflora* invasion, whereas the remaining households were not willing. Moreover, as per the inferential statistical results, there was a significant mean/percentage difference between nonwilling and willing households for the hypothesized variables, except for some variables such as farm experience, years living in the area, distance from the market, and dependency ratio.

After checking for estimation problems and applying remedies for the observed problems, the SUBP model results showed that a number of factors shape households' demand/willingness to mitigate *P. juliflora* invasion. Of the 17 variables included in the model, 4 of them, namely, sex, frequency of extension, contact, tenure, security, and livestock holding in TLU, presented a statistically significant and positive effect on the respondents' first response (WTP_1_). Contrary to this, the nonfarm income of the household head exhibited a statistically significant and negative effect on the respondents' first response (WTP_1_). Furthermore, five variables such as years living in the area, frequency of extension contacts, tenure, security, family size, and livestock holding in TLU were found to have a positive and significant effect on the second/follow-up response (WTP_2_). However, bid_2_, age, and nonfarm income of the household presented a negative and significant effect on respondents' second/follow-up response (WTP_2_).

### 5.2. Recommendations

Focusing on the general results and significant variables of the study, the following policy implications were recommended. The study recommends the need to boost the ability to eliminate cultural constraints and reduce the workload of female-headed households. This can be done by making female-headed households participate more in training, conferences, and gender mainstreaming programs, and there is a need to advise those female-headed households to marry another husband who can share the duty with them (which can be used to reduce their workload). Moreover, policymakers have to increase the chance that the household may live long in a particular area, and this can be done by increasing the opportunity of rural households to obtain forage for their livestock at the nearest distance. Even if households in the study area are mostly pastoral, their movement from place to place should have to be restricted. To do this, either the regional government or the national government should have to legislate a law that can restrict such movements. Furthermore, landowners should have to be given the right to play the chief role in controlling, managing, using, and transferring their own resources (land). To do so, owners of the land should have to be given the right to exclude others from using their resources without their permission and the right to transfer their ownership by self-interest to someone they want. Finally, the contact between extension agents and rural households should be strengthened; the frequency of their contacts should have to be increased; and the quality, practicality, and necessity of the services provided should have to be improved. This can be done by increasing the number of extension workers, training the existing workers, providing an incentive for those best performers, and punishing those kidding extension workers.

The findings of this study must be viewed in light of some potential limitations. First, the study was limited to Amibara and Awash Fentale districts only, which are selected randomly, and hence, taking samples from these districts only might not allow making generalization about the whole region. In addition to this, the study was also limited by the availability of published journal articles as related research was not conducted in ample generally in Ethiopia and especially in the study area and even WTP studies in any invasive alien species management as a general were also very rare. Natural resource valuation is not only important from an economic point of view and for natural resource conservation/improvement alone, but also for eradicating hazardous species from the environment (environmental management) and for harmonizing environmental management with community preferences. Due to these reasons and potential limitations, the study further recommends replicating similar investigations in the remaining regions where *P. juliflora* is introduced by using this finding as a benchmark.

## Figures and Tables

**Table 1:** Summary of measurement, type, and hypothesis of the variables.

Variables	Type of variables	Measurement	Hypothesis
*Dependent variables*			
WTP_1_/WTP_2_	Dummy	(1 if yes, 0 if no)	

*Independent variables*			
Initial bid (Bid_1_)	Continuous	Labor days	−ve
Follow-up bid (Bid_2_)	Continuous	Labor days	−ve
Age of the HH	Discrete	Number of years	−ve
Sex of the HH	Dummy	1 if male or 0 otherwise	+ve
Education level of the HH	Discrete	Number of years in school	+ve
Annual farm income	Continuous	Eth. birr (ETB^1^)	+ve
Non/off-farm income	Continuous	Eth. birr (ETB)	−/+
Security of tenure	Dummy	1 if secured or 0 otherwise	+ve
Past awareness	Dummy	1 if aware or 0 otherwise	+ve
Freq. of extension contact	Discrete	Number of days	+ve
Distance from the market	Continuous	Walking minutes	−ve
Farming experience	Discrete	Years	+ve
Years lived in the area	Continuous	Years	+ve
Size of own land	Continuous	Hectare	+ve
Family size	Continuous	Adult equivalent (AE)	−/+
Dependency ratio	Continuous	Numbers	−ve
Livestock holding	Continuous	Tropical livestock unit (TLU)	+ve

^1^ETB stands for Ethiopian birr, which is the official currency of the country.

**Table 2:** Descriptive statistics for continuous independent variables.

Variables	Willing (*n* = 293)		Nonwilling (*n* = 20)		*t* value
	Mean	Std. dev	Mean	Std. dev	
Age of the HH	44.41	10.69	58.20	7.00	5.68^*∗∗∗*^
Education level of the HH	0.32	1.33	2.15	3.41	5.13^*∗∗∗*^
Annual farm income	29016.88	20422.75	43595	19037.34	3.10^*∗∗∗*^
Non/off-farm income	4754.95	8506.59	24057.50	9349.81	9.76^*∗∗∗*^
Freq. of extension contact	5.23	4.69	0.65	1.18	−4.35^*∗∗∗*^
Distance from the market	101.57	121.58	73.65	29.27	−1.02
Farming experience	22.12	13.19	23.60	10.02	0.49
Years lived in the area	36.65	12.32	38.30	12.84	0.58
Size of own land	0.74	0.84	0.18	0.37	−2.99^*∗∗∗*^
Family size in AE	4.34	1.55	2.72	1.03	−4.59^*∗∗∗*^
Dependency ratio	1.29	1.04	1.09	1.01	−0.85
Livestock holding (TLU)	22.52	22.30	9.72	7.06	−2.56^*∗∗*^
Initial bid (Bid_1_)	9.30	5.70	14.20	4.30	3.77^*∗∗∗*^

^*∗∗∗*^ and ^*∗∗*^show significant variables at 1% and 5% probability levels, respectively. Source: own survey results, 2021.

**Table 3:** Descriptive statistics for dummy independent variables.

Variables	Category	Willing (*n* = 293)		Nonwilling (*n* = 20)		*χ* ^2^ value
		*N*	%	*N*	%	
Sex of the HH	Female	27	77.14	8	22.86	17.87^*∗∗∗*^
	Male	266	95.68	12	4.32	

Security of tenure	Secured	85	100.00	0	0.00	7.97^*∗∗∗*^
	Not secured	208	91.23	20	8.77	

Past awareness	Yes	211	98.14	4	1.86	23.55^*∗∗∗*^
	No	82	83.67	16	16.33	

^*∗∗∗*^shows significant variables at the 1% probability level. Source: own survey results, 2021.

**Table 4:** Determinants of rural households' WTP in *P. juliflora* management.

Variables	WTP_1_		WTP_2_		Joint marginal effect
	Coefficient	Robust std. err	Coefficient	Robust std. err	
Initial bid (Bid_1_)	−0.00753	0.0357			0.0000
Follow-up bid (Bid_2_)			−0.0443^*∗∗*^	0.0188	−0.0024
Age of the HH	−0.0422	0.0326	−0.0763^*∗∗∗*^	0.0158	−0.0041
Sex of the HH	0.8303^*∗*^	0.4327	−0.1471	0.3502	−0.0071
Education level of the HH	0.2226	0.0817	0.0958	0.0937	0.0052
Annual farm income (*ln*)	−0.3878	0.5964	0.4152	0.3431	0.0225
Non/off-farm income	−0.0000^*∗∗∗*^	0.0000	−0.0000^*∗∗∗*^	0.0000	−2.64*e*^−06^
Security of tenure	4.9436^*∗∗∗*^	1.0941	1.9497^*∗∗∗*^	0.5150	0.0705
Past awareness	0.2544	0.4870	0.2199	0.3386	0.0130
Freq. of extension contact	0.3802^*∗∗*^	0.1557	0.0732^*∗*^	0.0383	0.0040
Distance from the market	0.0011	0.0045	0.0029	0.0019	0.0001
Farming experience	0.0160	0.1793	−0.0023	0.0161	−0.0001
Years lived in the area	0.0080	0.0149	0.0380^*∗∗∗*^	0.0145	0.0021
Size of own land	−0.4137	0.2963	−0.1543	0.1717	−0.0083
Family size in AE	0.1200	0.1760	0.7786^*∗∗∗*^	0.1238	0.0421
Dependency ratio	−0.0803	0.2345	−0.1665	0.1496	−0.0090
Livestock holding (TLU)	0.0844^*∗∗∗*^	0.0267	0.0262^*∗∗∗*^	0.0100	0.0014

Cons	4.8324	6.1592	−3.7726	3.4970	

Number of observation = 313 Log-pseudo-likelihood = −79.33 Wald chi^2^ (32) = 664.97 Prob > chi^2^ = 0.0000		Rho = 0.729 Wald's test of rho = 0: chi^2^ (1) = 0.8004 Prob > chi^2^ = 0.0371 *y* = Pr (WTP_1_ = 1, WTP_2_ = 1) (predict, p_11_) = 0.977			

^*∗∗∗*^, ^*∗∗*^, and ^*∗*^show significant variables at 1%, 5%, and 10% probability levels, respectively. Source: own survey results, 2021.

## Data Availability

The data used to support the findings of this study are available from the corresponding author upon reasonable request.
